# Innovative Surgical Concept for Simon's Grade IIb Gynecomastia: A Systematic Integration of Circumareolar Mastectomy, Interlocking Suture, Inframammary Fold Detachment, and Waterjet-Assisted Liposuction for Superior Long-term Outcomes

**DOI:** 10.1093/asj/sjaf069

**Published:** 2025-05-02

**Authors:** Andreas Wolter, Sonia M Fertsch, Marc Daniels, Katrin Seidenstuecker, Beatrix Munder, Mazen Hagouan, Dirk Janku, Robert J Musmann, Christoph Andree, Dennis Hammond

## Abstract

**Background:**

Gynecomastia, particularly Simon's Grade IIb, poses aesthetic and psychological challenges because of glandular excess and skin redundancy. Standard circumareolar mastectomy (CM) often leads to hypertrophic scarring, areolar widening, and suboptimal contouring.

**Objectives:**

The authors of this study aim to evaluate a combined surgical approach integrating CM, circumareolar interlocking suture (CIS), inframammary fold detachment (IFD), and waterjet-assisted liposuction (WAL) in achieving improved aesthetic and functional outcomes for Simon's Grade IIb gynecomastia.

**Methods:**

A retrospective analysis was conducted on 95 patients (176 breasts) treated between April 2017 and December 2023. Of these, 72 patients (130 breasts) received the combined CM + CIS + IFD + WAL approach. Key outcomes included complication rates, nipple–areola complex (NAC) sensitivity, areolar diameter, and patient satisfaction assessed through the BODY-Q Chest and Nipples Scale. Mean follow-up was 17 months, with a subset followed up to 91 months.

**Results:**

The mean operative time was 85.1 min, and the average hospital stay was 2.75 days. The overall complication rate was 7.7%, with no cases of complete NAC necrosis. Areolar diameter significantly decreased postoperatively (*P* < .001). At 12 months, 71.2% of patients were “very satisfied” with scarring, and BODY-Q scores showed significant improvements in both chest and nipple domains. NAC sensation was preserved in 88.4% of cases.

**Conclusions:**

The CM + CIS + IFD + WAL technique offers a safe, effective, and aesthetically superior option for treating Simon's Grade IIb gynecomastia, with low complication rates and high patient satisfaction. Follow-up data suggest stable long-term outcomes, supporting the method's broader clinical relevance.

**Level of Evidence: 4 (Therapeutic):**

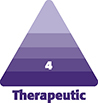

As a benign condition affecting male breast tissue, gynecomastia exhibits a range of appearances depending on body structure, making aesthetic correction challenging because of diverse patient expectations.^1^ Gynecomastia is notably common worldwide, with prevalence rates ranging from 32% to 65%.^2^ The causes of gynecomastia are diverse, but in over 80% of cases, the condition is idiopathic.^3,4^ This condition can profoundly affect psychological well-being, diminishing self-esteem and sexual identity, and often causing social embarrassment.^5-7^ Literature describes various classifications and surgical approaches that consider factors such as tissue predominance, breast size, the extent of breast ptosis, the vertical positioning of the nipple–areolar complex (NAC) relative to the inframammary fold (IMF), as well as skin elasticity, quality, and the degree of skin redundancy.^8-17^ In addition to the well-known traditional classification by Simon et al, the most recent and comprehensive system is the McMaster Classification by Waltho et al.^16,18^ For gynecomastia cases, Simon's Grade IIb or McMaster Grade IAii/IBii, characterized by periareolar skin redundancy, enlarged areola diameter, resection weight below 250 g, predominantly glandular, fibrous, or fatty tissue, ptosis Grade 0-I by Regnault, and the NAC positioned above the IMF, the preferred approach is often a scar-minimizing circumareolar mastectomy (CM), often combined with liposuction.^19^ The round-block technique, also known as the purse-string suture, as described by Benelli, has traditionally been used in similar cases, often alongside liposuction for Simon's Grade IIb gynecomastia;^20^ however, it frequently leads to hypertrophic scars that are not only prone to dehiscence but may also develop radiating extensions and contractile wrinkling around the areola, further compromising the aesthetic outcome.^21,22^ Originally developed for periareolar mastopexy in reduction mammaplasty and lifting procedures, the “circumareolar interlocking suture” was first introduced by Hammond et al.^23^ The “Benelli” technique relies on a purse-string suture that encircles only the outer boundary of the areola, creating a uniform but circumferential tension that often results in hypertrophic scarring.^24,25^ In contrast, the “interlocking suture” follows a wagonwheel pattern, incorporating longer stitches externally and shorter ones internally.^23^ This design ensures a more balanced distribution of tension, thereby reducing the risk of excessive contraction and improving aesthetic outcomes. The superiority of this technique in terms of tension redistribution has been physically validated in a study by Righi and Robotti, further supporting its effectiveness in minimizing hypertrophic scarring and optimizing wound healing.^26^ Our approach was refined by incorporating the circumareolar interlocking suture (CIS) alongside waterjet-assisted liposuction (WAL) and inframammary fold detachment (IFD), aiming to achieve optimal aesthetic results with minimal scarring. Drawing from our collective experience, we noted a remarkably low occurrence of hypertrophic or dehiscent scars around the areola, alongside high levels of patient satisfaction and minimal complication rates. The authors of this paper describe their surgical method in detail and present a retrospective analysis of their outcomes, comparing them with the existing literature.

## METHODS

This retrospective study included patients treated between April 2017 and December 2023 who underwent CM + CIS + IFD + WAL for Simon's Grade IIb gynecomastia. Patients were included if they had Simon's Grade IIb gynecomastia with periareolar skin redundancy, good-to-moderate skin elasticity, NAC positioned above the IMF, and resection weight <250 g, consistent with Regnault ptosis Grade 0-I. All patients underwent the combined CM + CIS + IFD + WAL technique. Patients were excluded if they presented with Simon's Grade I gynecomastia (treated through infra-areolar incision only), Grade III gynecomastia (requiring free or pedicled NAC transplantation), significant breast ptosis (Regnault ≥II), or poor skin quality. The preoperative evaluation included a comprehensive endocrinological analysis and a detailed clinical urological examination, with mammography of the affected breast performed in certain cases. Based on the algorithmic approach outlined in 2013, the choice of the most suitable surgical approach is influenced by multiple factors, including breast volume, the degree of breast ptosis, the vertical relation between the NAC and the IMF, NAC diameter, as well as skin elasticity, quality, and the degree of excess skin.^14^ All procedures were performed and monitored by 1 single surgeon (corresponding author) throughout the study period. In gynecomastia cases of Simon's Grade IIb, we implemented the CM, CIS, IFD, and WAL approach.^18^ The collected dataset included patient-related factors (Table 1), such as age, BMI, smoking status, and breast measurements, including NAC diameter before and after surgery. Additionally, glandular tissue resection weight, duration of hospitalization, operative time, and key outcome measures (Table 2), such as complication rates and the necessity for surgical revisions, were documented. Patient satisfaction was additionally evaluated using the Chest and Nipples Scale of the BODY-Q questionnaire, a validated, internationally recognized patient-reported outcome measure (PROM) specifically designed for postbody contouring procedures, supplemented by a subjective assessment of nipple sensitivity on a scale from 1 (very sensitive) to 4 (no sensitivity).^27^ Authorization to utilize the German version of the BODY-Q Chest module was granted by the copyright holders, with the questionnaire having previously undergone translation and linguistic validation.^28^ Overall scores were converted into Rasch scores, with a range from 0 (worst) to 100 (best). Satisfaction with postoperative scarring was assessed using the BODY-Q Chest and Nipples Scale, rated on a 4-point scale ranging from “very dissatisfied” to “very satisfied.” The BODY-Q Chest and Nipples questionnaires were completed on paper, either in person during the follow-up visit or through telephone interview for patients unable to attend. The survey was not anonymous because it was linked to patient-specific follow-up data, but all responses were handled confidentially and in accordance with ethical standards. Distribution and collection were conducted by the corresponding author. Standardized perspective photographs were captured for all patients pre- and postoperatively.

**Table 1.** Patient Demographics

Demographic details	*n* (%)	
Total no. of patients/mastectomies	95/176	
Of these circumareolar (CM combined with CIS, WAL and IFD)	72/130 (73.9%)	
Unilateral/bilateral	14 (19%)/58 (81%)	
Smokers	8 (11.1%)	
Idiopathic cause of gynecomastia	70 (97.2%)	
Drug-induced cause of gynecomastia	2 (2.8%)	

	Mean/SD	Range
Follow-up time (months)	16.99 ± 11.46	12-91
Age (years)	32.47 ± 13.37	18-77
BMI (kg/m^2^)	25.5 ± 2.76	20.8-35.9
BMI categorical		
Underweight (<18.5 kg/m^2^)	0 pts (0%)	
Normal (18.5-24.9 kg/m^2^)	33 pts (45.8%)	
Overweight (25-29.9 kg/m^2^)	35 pts (48.6%)	
Obese (>30 kg/m^2^)	4 pts (5.6%)	
Distance between sternal notch and NAC in cm (right)	19.64 ± 1.60	17-23
Distance between sternal notch and NAC in cm (left)	19.68 ± 1.67	17-23
Distance from NAC to IMF in cm (right)	3.69 ± 1.17	2.0-7
Distance from NAC to IMF in cm (left)	3.72 ± 1.22	2.0-7
Breast width in cm (right)	18.10 ± 2.14	12-24
Breast width in cm (left)	18.28 ± 2.43	12-26
NAC diameter in mm (right) preop	33.21 ± 4.87	22-45
NAC diameter in mm (right) postop	26.89 ± 2.85*	19-37
NAC diameter in mm (left) preop	33.26 ± 4.95	23-43
NAC diameter in mm (left) postop	27.13 ± 2.68*	20-35
Underbust circumference (cm)	84.07 ± 7.38	71-105
Breast resection weight in g (right)	41.0 ± 46.80	4-250
Breast resection weight in g (left)	50.14 ± 53.73	2-280
Lipoaspirate volume in mL (right)	153.19 ± 131.41	20-600
Lipoaspirate volume in mL (left)	171.82 ± 156.34	30-700
Operative time (min)	85.10 ± 41.05	20-185
Hospital stay (days)	2.75 ± 1.4	1-7
Breast skin elasticity		
Good	117/130 (89.7%)	
Intermediate	10/130 (8%)	
Poor	3/130 (2.3%)	

CIS, circumareolar interlocking suture; CM, circumareolar mastectomy; IMF, inframammary fold; NAC, nipple–areolar complex; SD, standard deviation; WAL, waterjet-assisted liposuction. *P < .001, paired *t*-test for continuous variables, *χ*^2^ test, or Fisher’s exact test for categorical variables.

**Table 2.** Outcome Parameters (72 Patients, 130 Breasts/Mastectomies)

Overall complications	10 (7.7%)	
Minor	6 (4.6%)	
Partial NAC necrosis	2 (1.5%)	
Seroma needle aspiration	2 (1.5%)	
Wound dehiscence healed by secondary intention	2 (1.5%)	
Major	4 (3.1%)	
Full NAC necrosis	0 (0%)	
Acute hematoma with surgical evacuation	3 (2.3%)	
Wound dehiscence with surgical repair	1 (0.8%)	
Secondary revisions/corrections	5 (3.8%)	
NAC revisions	3 (2.3%)	
Reoperation because of recurrence	2 (1.5%)	
Nipple sensitivity		
(1) Very sensitive	69/112 (61.6%)	
(2) Sensitive	30/112 (26.8%)	
(3) Less sensitive	10/112 (8.9%)	
(4) Not sensitive	3/112 (2.7%)	
BODY-Q—Chest and Nipples Scales		
Nipples		
Preoperative	33.85 ± 19.2	
Postoperative	76.3 ± 9.19	*P* < .001*
Chest		
Preoperative	25.33 ± 12.09	
Postoperative	84.83 ± 7.5	*P* < .001*
Satisfaction with appearance of the scars		
Very satisfied	42/59 (71.2%)	
Somewhat satisfied	15/59 (25.4%)	
Somewhat dissatisfied	2/59 (3.4%)	
Very dissatisfied	0/59 (0%)	

NAC, nipple–areolar complex. *P < .001, paired *t*-test for continuous variables, *χ*^2^ test, or Fisher's exact test for categorical variables.

### Statistical Evaluation

Continuous data were expressed as mean ± standard deviation, whereas categorical data were summarized as absolute and relative frequencies. To compare continuous variables before and after surgery, a paired *t*-test was applied. Categorical variables were analyzed using either the *χ*^2^ test or Fisher's exact test, depending on applicability. A *P*-value of <.05 was considered statistically significant. All statistical calculations, including means, standard deviations, and *t*-tests, were conducted using Microsoft Excel for Mac Version 16.7 (Microsoft Corporation, Redmond, WA) and SPSS Statistics Version 29.0.0.0 (IBM Statistics, Chicago, IL).

### Ethical Compliance

This retrospective study adhered to the latest ethical principles outlined in the Declaration of Helsinki and followed the guidelines set by the International Committee of Medical Journal Editors. Ethical approval, including consent procedures, was granted by the Ethical Board Committee of the University of Witten/Herdecke, Faculty of Health, Witten, Germany, under File Decision Number S-002-/2024. All data were anonymized and examined retrospectively.

### Operative Approach

**Figure 1.**
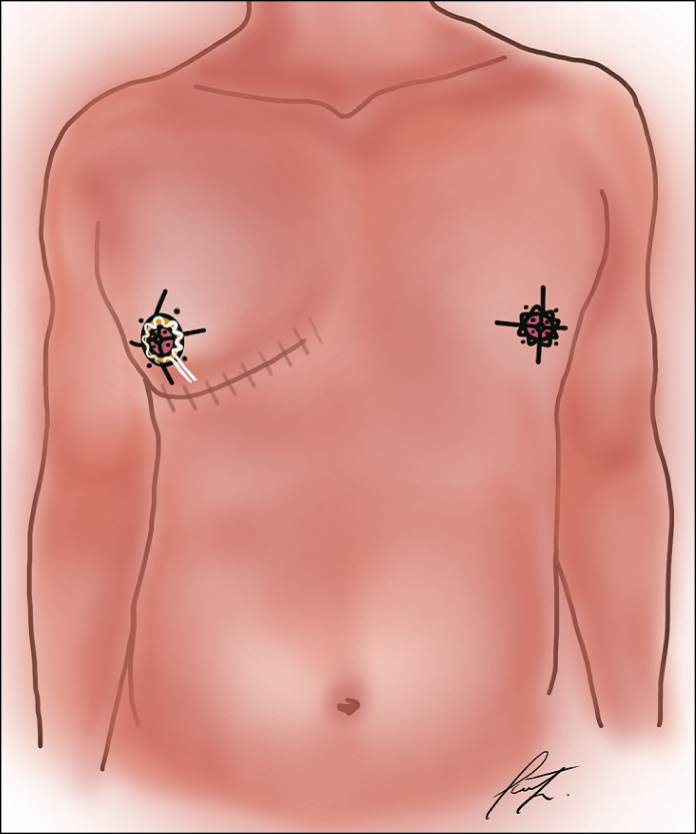
Illustration of chest wall contouring technique. The markings on the patient's right nipple illustrate the circumareolar interlocking suture (CIS) and inframammary fold detachment (IFD; shaded area). The markings on the patient's left nipple illustrate the status after CIS with reduction of nipple–areolar complex (NAC) diameter, slight craniolateral elevation of NAC position, and IFD emphasizing the contour of the pectoralis major muscle.

**Figure 2.**
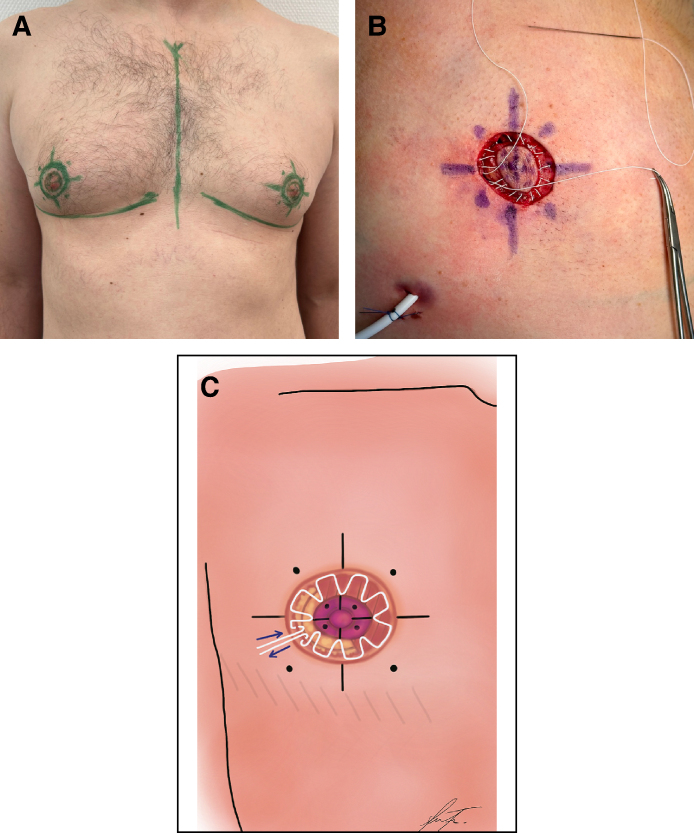
The circumareolar interlocking suture (CIS) technique demonstrated in a 52-year-old male patient. (A) Markings were made using a surgical pen, dividing the outer circle evenly at the main clock positions: 12, 3, 6, and 9 o’clock, with additional points halfway between these (eg, 2:30 and 10:30). (B) The inner nipple–areolar complex (NAC) was similarly divided into quarters, aligning with these outer circle points. A lateral incision for the mastectomy was performed, ensuring the superomedial pedicle supporting the NAC remained intact. To adjust the NAC position as needed, a cranio-oval deepithelialization was performed, ideally aligning with the pectoralis muscle. The CIS begins by threading the white PTFE thread from deep to superficial (blue arrows) through the de-epithelialized edge, then looping back from superficial to deep, allowing the knot to be securely buried beneath the skin to minimize postoperative exposure. (C) Finally, inframammary fold detachment was performed from the inner chest cavity to eliminate the characteristic female breast contour, as shown in the shaded area of the illustration.

**Figure 3.**
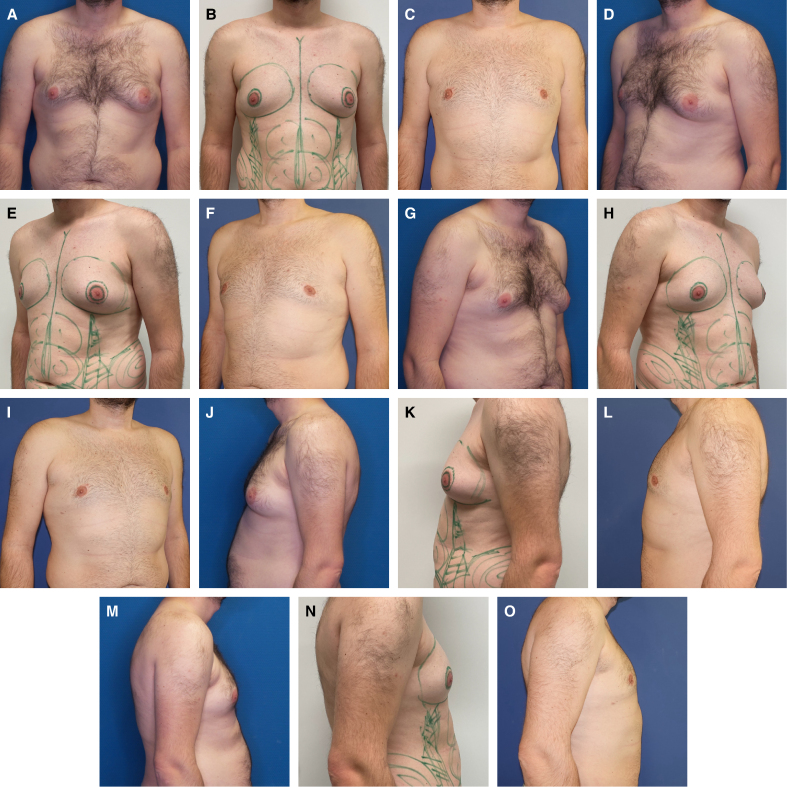
(A, D, G, J, M) A 36-year-old male patient preoperatively with ptosis Grade 0, SN-NAC 20 cm on the right side, 21 cm on the left side, NAC-IMF 4 cm on the right side, 6 cm on the left side, breast width 13 cm on the right side, 14 cm on the left side, NAC diameter 32 cm on the right side, 42 cm on the left side, underbust circumference 86 cm, and BMI of 26.8 kg/m^2^. (B, E, H, K, N) Photographs depicting the patient with preoperative markings. (C, F, I, L, O) Photographs depicting the patient 42 months postoperatively, resection weight was 42 g on the right side, 140 g on the left side, 150 mL of lipoaspirate on the right side, and 250 mL of lipoaspirate on the left side. IMF, inframammary fold; NAC, nipple–areola complex.

Breast assessments, preoperative markings, and surgical procedures were conducted by the corresponding author both before and following the operation. The CIS figure, along with the IMF, was marked on standing patients using moderate pinching (Figures 1, 2; Video 1). The planned NAC was outlined with an inner circular marking, measuring between 2.0 and 3.0 cm in diameter. The outer periareolar boundary was adapted according to the degree of excess skin present. To ensure an anatomically suitable positioning on the chest, a superiorly elongated oval excision was performed, as demonstrated in the patient example (Figure 3). A single perioperative dose of antibiotics was administered to all patients, along with 1 g of tranexamic acid to minimize intraoperative bleeding.

Hydrodissection of the breast gland was carried out using WAL with the BODY-JET system from Human Med AG (Wilhelm–Hennemann–Strasse, Schwerin, Germany; Video 1). For tumescence, a mixture containing 1000 mL of Ringer's solution and 1 mL of 1:1000 diluted adrenaline was administered. Liposuction was performed in both the subcutaneous and epipectoral planes before the mastectomy to facilitate tissue separation, promote vasoconstriction, and refine the contours of the surrounding subcutaneous fat, contributing to a more masculinized chest shape. The cannulas used for liposuction had diameters ranging from 3.5 to 4.2 mm.

**Figure 4.**
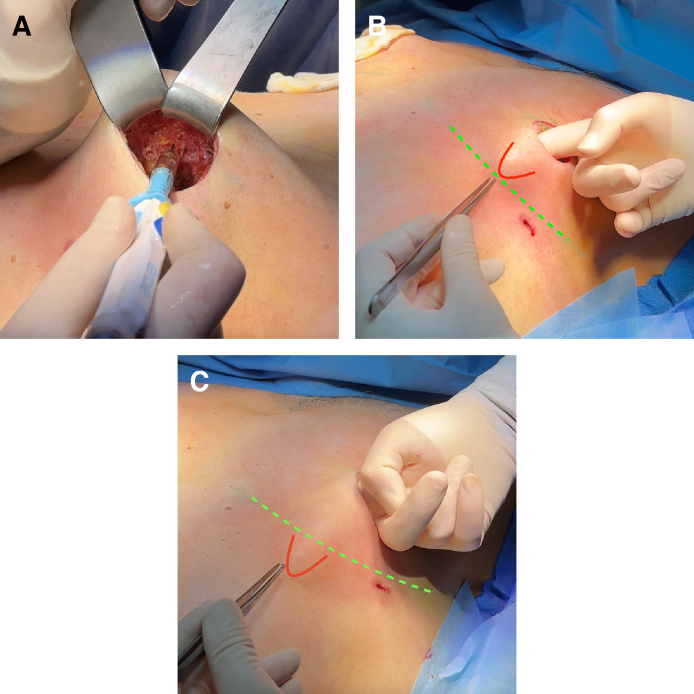
Inframammary fold detachment (IFD) technique. (A) Intraoperative view of the breast cavity in a 52-year-old male patient, transection of Cooper's ligaments using monopolar electrocautery. (B) Demonstration of the inframammary fold (IMF; green dashed line) before IFD (red outline at the fingertip, extending to the intact IMF within the internal breast cavity. (C) Demonstration of the released (detached) IMF (green dashed line) after IFD (red outline at the fingertip, extending caudally beyond the transected IMF).

To perform the CM, the skin around the NAC was circumferentially deepithelialized, outlining its outer margin (Figures 1, 2; Video 2). A lateroareolar incision was used to access the breast gland, ensuring optimal preservation of the superomedial pedicle's neurovascular supply. For enhanced visibility, a headlight was utilized during the procedure. The mammary gland was meticulously dissected in the subcutaneous and epifascial planes with the aid of electric cautery, maintaining Cooper's ligaments and ensuring a pedicle thickness of ∼1.5 to 2 cm. To ensure optimal chest contouring, emphasis was placed on IFD for the release of restrictive fascial bands (Figure 4). Following excision, the breast cavity was rinsed with saline, and 1 g of tranexamic acid was applied locally. Drains were placed at the liposuction entry points and removed when the secretion volume dropped below 30 mL within 24 h. Finally, all removed breast tissue was sent for histopathological examination.

The CIS was first outlined using a surgical marker before suturing (Figures 1, 2; Video 2). A 2-0 PTFE nonabsorbable suture (SurgiGLIDE, SurgiGlide LLC, Grand Rapids, MI) with a Keith needle was used, ensuring the areolar diameter was adjusted between 20 and 30 mm. To reduce the risk of postoperative exposure, the suture knot was embedded beneath the periareolar incision. The final closure of the areola was performed intracutaneously using 4-0 Monocryl. Postoperatively, wounds were covered with fatty gauzes and compresses. A compression bandage was applied for the first 24 h, after which patients transitioned to wearing a compression vest for 6 weeks. To support healing, manual lymphatic drainage of the chest was recommended once per week, starting 2 weeks postoperatively and continuing for 6 to 8 weeks. Patients were also advised to avoid strenuous upper body exercise for a total of 6 months.

## RESULTS

**Figure 5.**
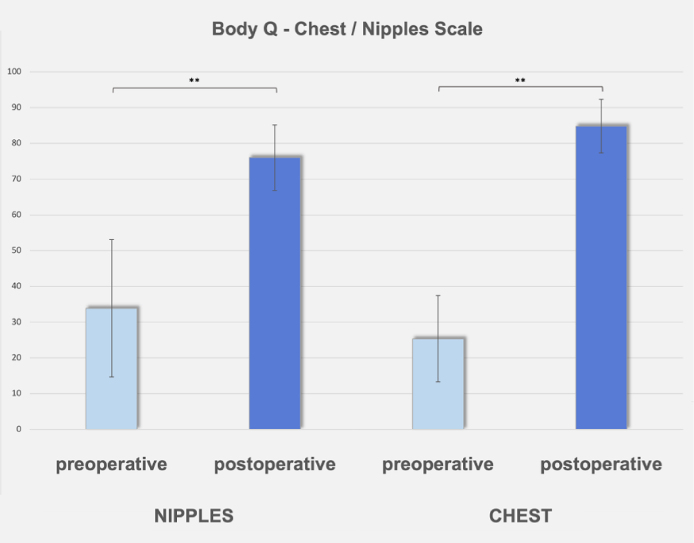
BODY-Q Chest and Nipples Scales. The BODY-Q results, comparing preoperative scores to those at 12 months postoperatively, showed a statistically significant improvement in the Rasch sum scores for both the Chest and Nipples Scales. The Nipples Scale score increased from 33.85 ± 19.2 preoperatively to 76.3 ± 9.19 postoperatively (**P < .001), whereas the Chest Scale score improved from 25.33 ± 12.09 to 84.83 ± 7.5 (**P < .001).

From April 2017 to December 2023, a total of 176 mastectomies were performed on 95 patients diagnosed with gynecomastia. Among them, 72 individuals (130 breasts, 73.9%) with Simon's Grade IIb underwent treatment using a combination of CM, CIS, IFD, and WAL. The remaining patients received either an infra-areolar approach for Simon's Grade I or an inframammary technique, with free or pedicled NAC transplantation for those classified as Simon's Grade III. Bilateral gynecomastia was present in 58 patients (81%), whereas 14 patients (19%) exhibited a unilateral form of the condition. Eight patients (11.1%) reported active smoking at the time of surgery. In 70 cases (97.2%), gynecomastia was classified as idiopathic, whereas in 2 cases (2.8%), it was associated with medication use—1 linked to neuroleptic treatment and the other to excessive cannabis consumption. Patients ranged in age from 18 to 77 years, with a mean age of 32.5 ± 13.4 years, as detailed in Table 1. The mean BMI recorded was 25.5 ± 2.76 kg/m^2^. Analysis of BMI distribution showed that none of the patients fell into the underweight category, whereas 45.8% (33 patients) had a normal BMI, 48.6% (35 patients) were classified as overweight, and 5.6% (4 patients) were considered obese. Breast elasticity assessment indicated that 89.7% (117 of 130 breasts) had “good” elasticity, 8% (10 of 130) showed “intermediate” elasticity, and 2.3% (3 of 130) were classified as “poor.” The mean distance measured from the sternal notch to the NAC was 19.64 ± 1.60 cm on the right side and 19.68 ± 1.67 cm on the left. Furthermore, the average NAC-to-IMF distance was recorded at 3.69 ± 1.17 cm on the right and 3.72 ± 1.22 cm on the left. The width of the breasts measured on average 18.10 ± 2.14 cm on the right and 18.28 ± 2.43 cm on the left, whereas the preoperative NAC diameter was determined to be 33.21 ± 4.87 mm on the right and 33.26 ± 4.95 mm on the left. The mean underbust circumference was 84.07 ± 7.38 cm. Patients remained hospitalized for an average of 2.75 ± 1.4 days. The mean duration of surgery was 85.10 ± 41.05 min. The average mass of excised breast tissue was 41.0 ± 46.80 g on the right and 50.14 ± 53.73 g on the left. Histopathological evaluation did not reveal any malignant or pathological alterations. The total volume of lipoaspirate removed averaged 153.19 ± 131.41 mL on the right side and 171.82 ± 156.34 mL on the left. The overall complication rate was 7.7%, affecting 10 breasts (Table 2). Major complications requiring surgical intervention occurred in 4 cases (3.1%). The most frequent major complication was acute hematoma, necessitating surgical evacuation in 3 breasts (2.3%), whereas 1 case (0.8%) of wound dehiscence required operative correction. In contrast, minor complications, which were managed conservatively, were observed in 6 mastectomies (4.6%). These included seroma, requiring needle aspiration in 2 cases (1.5%), partial necrosis of the NAC in another 2 cases (1.5%), and wound dehiscence that healed secondarily in 2 patients (1.5%). Notably, there were no cases of complete NAC necrosis (0%). Secondary surgical interventions were needed in 5 cases (3.8%) for revision procedures, including NAC modifications such as concentric retightening and areolar reshaping in 3 instances (2.3%), as well as reoperation for recurrence in 2 cases (1.5%). There was no case requiring a secondary liposuction. To ensure consistency in postoperative evaluations, all patients were scheduled for follow-up examinations no earlier than 12 months after surgery. The follow-up period ranged from 12 to 91 months, with a mean of 16.99 ± 11.46 months and a median of 12 months. A total of 59 patients (82%) completed preoperative questionnaires and were reassessed either in person at their 12-month follow-up or through telephone interviews. Regarding scar satisfaction, 71.2% of patients (42 out of 59) reported being “very satisfied” with the periareolar and stab incision/drain scars, whereas 25.4% (15 out of 59) expressed being “somewhat satisfied.” Only 3.4% (2 out of 59) were “somewhat dissatisfied,” and no patients (0%, 0 out of 59) described their satisfaction level as “very dissatisfied.” A total of 59 participants provided subjective evaluations of their nipple sensitivity, assessing 112 NACs, including 53 cases bilaterally and 6 unilaterally. Sensation levels were categorized as follows: 69 NACs (61.6%) were described as “very sensitive” (1), 30 NACs (26.8%) as “sensitive” (2), 10 NACs (8.9%) as “reduced sensitivity” (3), and 3 NACs (2.7%) were reported as “insensitive” (4). A comparison of NAC diameters before and after surgery revealed a statistically significant decrease in areolar dimensions. Following surgery, a notable reduction in NAC diameter was recorded. On the right side, the average measurement declined from 33.21 ± 4.87 mm before surgery to 26.89 ± 2.85 mm postoperatively (*P* < .001). Similarly, the left NAC shrank from a preoperative mean of 33.26 ± 4.95 to 27.13 ± 2.68 mm after the procedure (*P* < .001). Patients expressed a high degree of satisfaction with the aesthetic results achieved. When BODY-Q evaluations conducted before surgery were compared with data collected at the 12-month follow-up, a substantial increase in the Rasch sum score for the Chest and Nipples Scales was evident. The Nipples Scale demonstrated a marked improvement, rising from a baseline score of 33.85 ± 19.2 to 76.3 ± 9.19 postoperatively (*P* < .001). Likewise, the Chest Scale showed significant progress, with scores increasing from 25.33 ± 12.09 preoperatively to 84.83 ± 7.5 at the 1-year follow-up (*P* < .001; Table 2, Figure 5).

## DISCUSSION

The enlargement of male breast tissue, known as gynecomastia, is a benign condition that can significantly impact both psychological well-being and social confidence, necessitating advancements in surgical techniques to optimize both functional and aesthetic outcomes. The authors of this study present the CIS in combination with CM, IFD, and WAL as a refined surgical approach designed to enhance long-term results while minimizing visible scarring. Classification systems originally developed by Simon et al, and subsequently modified by various researchers, have proven instrumental in guiding treatment selection.^12,15,16,18^ As demonstrated in earlier studies and reflected in our cohort (73.9%), Simon's Grade IIb represents the most common category among individuals seeking surgical correction.^29^ CM is predominantly indicated for patients with Simon's Grade IIb, particularly in cases where skin elasticity and quality are favorable, and the NAC is positioned above the IMF, aligning with algorithmic treatment recommendations previously established.^14-18,30^

### Limitations of the Conventional Circumareolar Approach

Originally described by multiple researchers in the late 1990s, the circumareolar purse-string technique continues to be a frequently employed surgical approach in modern clinical practice.^21,31-33^ The CM technique relies on a periareolar incision to achieve 2 primary objectives. Firstly, it facilitates direct access to the breast tissue, enabling efficient glandular excision while simultaneously preserving nipple sensitivity and minimizing visible scarring. Secondly, excising skin around the areola helps reduce the overall skin envelope dimensions in cases where significant excess glandular tissue is accompanied by a larger volumetric component. Despite these benefits, the restricted surgical access provided by CM may, in certain patients, limit the extent of tissue removal, potentially compromising aesthetic results, increasing the likelihood of gynecomastia recurrence, and necessitating revision procedures. The periareolar incision has been statistically linked to a greater incidence of complications than other incision techniques and is more prone to causing conspicuous pathological scarring.^34-39^ Excessive outer skin resection can contribute to NAC deformation during glandular excision and subsequent wound closure, potentially resulting in complications, such as a flattened nipple contour, tissue wrinkling, an enlarged areolar diameter, or the development of thickened and uneven scarring, which may later necessitate secondary corrective procedures.^22,40^

### Advancements in Circumareolar Surgery: CIS, IFD, and WAL Integration

**Figure 6.**
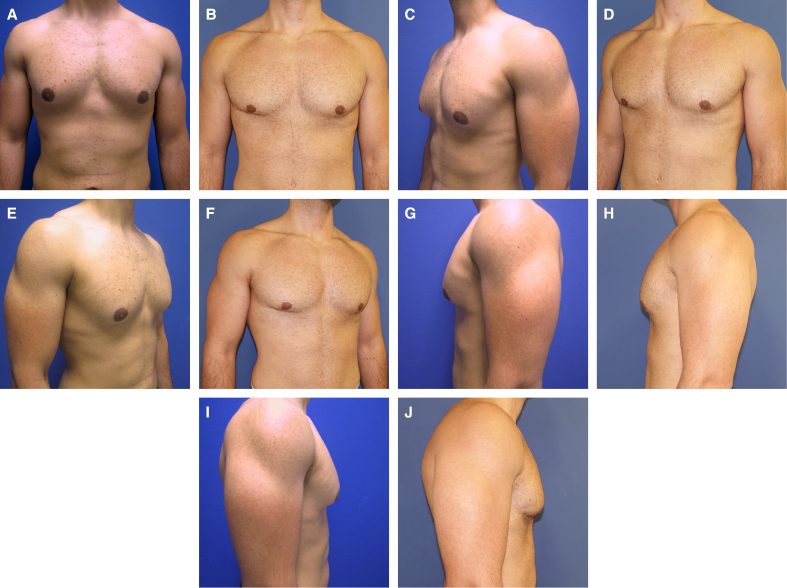
(A, C, E, G, I) A 30-year-old male patient preoperatively in standard perspectives, with ptosis Grade 0, SN-NAC 21 cm on both sides, NAC-IMF 3.1 cm on both sides, breast width 19 cm on both sides, NAC diameter 4.7 cm on both sides, underbust circumference 85 cm, and BMI of 24.1 kg/m^2^. (B, D, F, H, J) Photographs depicting the patient 91 months postoperatively, with a resection weight 22 g on the right side, 25 g on the left side, and 50 mL of lipoaspirate on each side. IMF, inframammary fold; NAC, nipple–areola complex.

**Figure 7.**
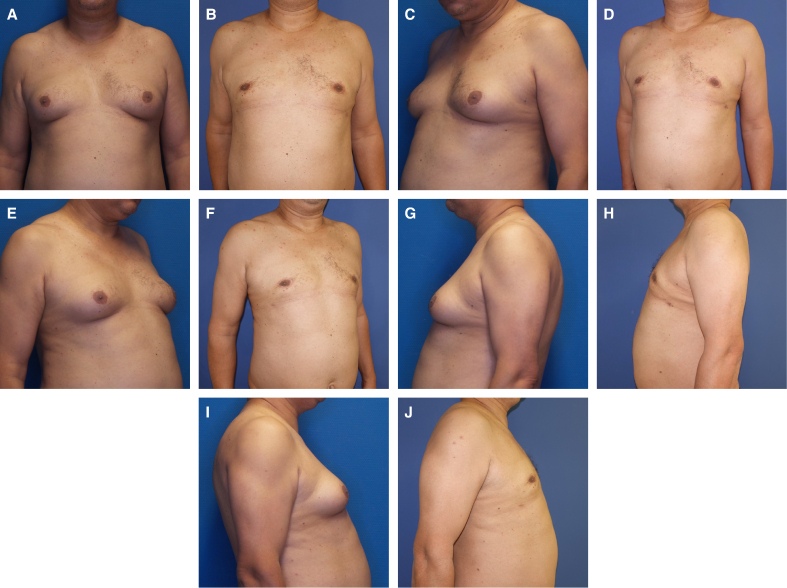
(A, C, E, G, I) A 46-year-old male patient preoperatively in standard perspectives, with ptosis Grade 1, SN-NAC 20 cm on both sides, NAC-IMF 7 cm on both sides, breast width 17 cm on both sides, NAC diameter 3.7 cm on the right side, 4.0 cm on the left side, underbust circumference 105 cm, and BMI of 31.9 kg/m^2^. (B, D, F, H, J) Photographs depicting the patient 26 months postoperatively. Resection weight was 62 g on the right side, 65 g on the left side, 300 mL of lipoaspirate on right side, and 500 mL of lipoaspirate on left side. IMF, inframammary fold; NAC, nipple–areola complex.

**Figure 8.**
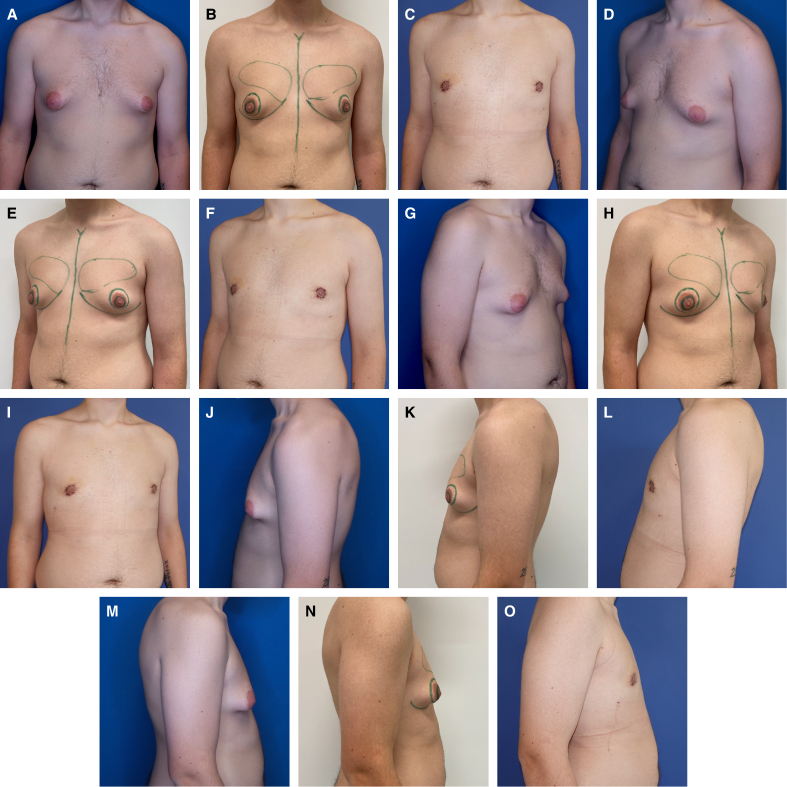
(A, D, G, J, M) A 20-year-old male patient preoperatively in standard perspectives, with areolar prolapse on both sides, ptosis Grade 0, SN-NAC 22 cm on right side, 22 cm on left side, NAC-IMF 5 cm on both sides, breast width 16 cm on both sides, NAC diameter 4.1 cm on the right, 4.2 cm on the left, underbust circumference 83 cm, and BMI of 25.4 kg/m^2^. (B, E, H, K, N) Photographs depicting the patient with preoperative markings. (C, F, I, L, O) Photographs of the paitent 3 weeks postoperatively, with a resection weight of 95 g on the right side, 85 g on the left side, and 50 mL of lipoaspirate on each side. Note the craniolateral elevation of the NAC position, highlighted by the green lines placed on anatomically identical regions. IMF, inframammary fold; NAC, nipple–areola complex.

**Figure 9.**
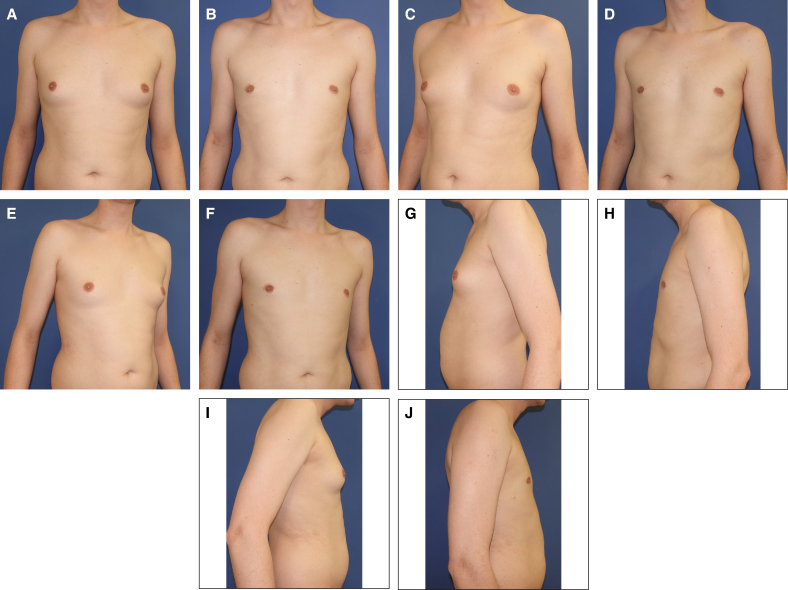
(A, C, E, G, I) A 31-year-old male patient preoperatively in standard perspectives, with ptosis Grade 0, SN-NAC 18 cm on right side, 18 cm on left side, NAC-IMF 6 cm on both sides, breast width 14 cm on both sides, NAC diameter 4.0 cm on the right, 3.7 cm on the left, underbust circumference 85 cm, and BMI of 21.4 kg/m^2^. (B, D, F, H, J) Photographs of the patient 19 months postoperatively, with a resection weight of 62 g on the right side, 58 g on the left side, and 200 mL of lipoaspirate on each side. IMF, inframammary fold; NAC, nipple–areola complex.

The combination of CM with CIS, IFD, and WAL was developed to mitigate or even entirely prevent these potential complications when utilizing a circumareolar approach for patients with Simon's Grade IIb gynecomastia. Initially introduced by D.H. for periareolar breastlifting in female patients, the “interlocking suture” presents a distinct advantage by ensuring a more uniform distribution of circumareolar tension when compared with conventional purse-string sutures, a benefit that has been experimentally validated by Righi and Robotti.^23,26^ Although the mean follow-up duration in our study was 16.99 ± 11.46 months, a subset of patients was monitored for up to 91 months, allowing preliminary insights into the technique's long-term effects. Importantly, among patients followed for >36 months, results remained stable over time, indicating that CIS supports sustained tissue adaptation. However, further prospective studies with extended follow-up periods will be necessary to validate these findings and assess possible late-stage volume fluctuations. Patient-reported satisfaction levels were notably very high (BODY-Q Chest Scale: 76.3 ± 9.19), with only 3.4% of individuals expressing moderate dissatisfaction regarding scarring, suggesting a potential benefit of the CIS approach (Table 2). A crucial component of our technique is the optimization of the IMF contour, accomplished through IFD (Figure 4). By strategically severing restrictive ligaments within the superficial fascial system, this method enables seamless adaptation of the thoracic skin to the underlying chest wall, ensuring a more natural and aesthetically refined transition (Figures 3, 6-9).

### Patient-Reported Outcomes and Aesthetic Perception

The integration of PROMs in this study provides valuable insight into the subjective patient experience, complementing the objective aesthetic outcomes achieved through surgical refinement—particularly regarding the IMF contouring. Although the IMF often receives less attention in traditional outcome reporting, it plays a critical role in overall chest definition and masculinization, especially in Simon's Grade IIb gynecomastia. Our findings suggest that targeted release of fascial bands at the IMF through IFD contributes not only to technical success but also to heightened patient-reported satisfaction, as evidenced by the significant improvements in BODY-Q Chest and Nipples Scale scores (Figure 5). This correlation between the refined IMF approach and improved PROM scores may underscore the psychological and aesthetic importance of achieving a natural, smooth transition from the chest wall to the upper abdomen. Because this area is readily visible and prone to postoperative contour irregularities, a deliberate IMF technique likely has a disproportionately positive impact on patient-perceived symmetry and body image—both core elements measured in BODY-Q domains. Therefore, PROMs not only validate surgical outcomes but also reinforce the importance of addressing less-emphasized anatomical zones such as the IMF in gynecomastia correction. Research studies on gynecomastia that incorporate internationally standardized and widely accepted PROMs, such as the BREAST-Q or the BODY-Q Chest and Nipples Module applied in this study, remain scarce in the existing literature.^27,40-43^ In line with previous findings, our results demonstrate an exceptionally high level of patient satisfaction regarding aesthetic outcomes (Table 2). The survey response rate was notably high, with 59 out of 72 patients (82%) participating. The rate of NAC revisions in our study was remarkably low at 2.3%, encompassing both NAC adjustments and contour corrections—an important finding given that conventional purse-string techniques in CMs have historically been linked to poor scarring and NAC distortion in up to 15% of cases during follow-up, often as a result of radial scar extensions.^7,25,31,44^ Unlike the conventional caudal entry, our approach involves lateral access with a superomedial pedicled nipple (Figures 1, 2), improving anatomical preparation and ensuring greater neurovascular preservation. This technique enhances the safety of the neurovascular supply, contributing to a remarkably low rate of related complications. By employing a lateral incision and superomedial pedicle method, we successfully maintained NAC sensitivity, with 88.4% of patients describing their sensation as either “very sensitive” or “sensitive,” and, to the best of our knowledge, this approach has not yet been documented in periareolar gynecomastia surgery. Furthermore, the total complication rate remained low at 7.7%, with only 2.3% of patients experiencing acute postoperative bleeding and no cases of complete NAC necrosis. The WAL method demonstrated multiple benefits, particularly in the context of gynecomastia surgery. Its hydrodissection capability allowed for highly controlled and efficient glandular tissue extraction, which is especially advantageous given the firm and fibrous consistency of male breast tissue. Additionally, the infusion of adrenaline during the procedure significantly reduced intraoperative bleeding, whereas its secondary effect contributed to mitigating postoperative inflammatory responses, enhancing overall surgical outcomes.^45^ Moreover, in obese patients, WAL facilitates precise contouring of the chest wall and liposculpting, improving aesthetic outcomes while minimizing overall tissue trauma.^46^ Regarding the superomedial pedicle: in our cohort, postoperative prominence of the preserved pedicle was not observed. This is likely because of the combination of targeted WAL in the epipectoral and subcutaneous layers and meticulous contouring during resection. Additionally, the lateral access approach allows for precise shaping of the transition zone, further minimizing bulging.

### Study Limitations and Future Research

A possible limitation of this study is its retrospective design, which could introduce biases in data collection. Additionally, the relatively short average follow-up period may restrict the ability to fully evaluate long-term functional and aesthetic outcomes. Another limitation lies in the absence of a prospective control group or randomized comparison with alternative surgical techniques, such as the traditional purse-string method, inframammary approaches, or liposuction-only strategies. This restricts the generalizability of our findings and prevents direct benchmarking against established standards. The study cohort was demographically homogeneous, with a relatively narrow age range and predominantly idiopathic gynecomastia cases. Future research should aim to include more diverse populations in terms of age, ethnicity, gynecomastia etiology, and comorbidities to better assess broader applicability. Although the BODY-Q is a validated tool, reliance on self-reported satisfaction may be influenced by individual expectations and recall bias. Future studies should combine PROMs with objective surgeon-rated aesthetic outcome scales to enhance validity. Although some patients were followed for up to 91 months, the average follow-up duration of 17 months limits insights into very long-term outcomes, such as scar maturation, late recurrence, or changes in nipple sensitivity over time. A key advantage of this study lies in the remarkable homogeneity of its cohort, comprising 72 patients who collectively underwent 130 CM procedures for gynecomastia. Moreover, to maintain uniformity in both surgical technique and postoperative assessment, a single surgeon (corresponding author) was responsible for performing all operations and follow-up examinations, ensuring consistency across the entire study population. Considering the significant influence of gynecomastia surgery on self-perception and overall well-being, extensive future research with larger patient cohorts will be essential to substantiate the effectiveness of this approach and assess its potential for wider clinical implementation.

## CONCLUSIONS

The integration of CM with CIS, IFD, and WAL introduces an advanced surgical strategy for managing Simon's Grade IIb gynecomastia. Our results indicate a high degree of patient satisfaction, well-preserved nipple sensitivity, and a notably low incidence of complications. Moreover, the technique appears to provide stable long-term outcomes, reinforcing its potential as an effective and reliable treatment option. Although further studies are needed to validate these results across diverse patient groups, the technique offers a valuable alternative with superior aesthetic and functional outcomes.

## Supplemental Material

This article contains supplemental material located online at https://doi.org/10.1093/asj/sjaf069.

## Supplementary Material

sjaf069_Supplementary_Data
